# Differential Impacts of Extreme Weather Events on Vector-Borne Disease Transmission Across Urban and Rural Settings: A Scoping Review

**DOI:** 10.3390/healthcare13192425

**Published:** 2025-09-25

**Authors:** Ahmad Y. Alqassim

**Affiliations:** Family and Community Medicine Department, Faculty of Medicine, Jazan University, Jazan 45142, Saudi Arabia; aalqassim@jazanu.edu.sa

**Keywords:** climate change, vector-borne diseases, urban health, rural health, extreme weather events, disease prevention, healthcare preparedness, malaria, dengue, scoping review study

## Abstract

**Background/Objectives**: Climate change is intensifying vector-borne disease (VBD) transmission globally, causing over 700,000 annual deaths, yet systematic evidence comparing climate–health pathways across urban and rural settlements remains fragmented. This scoping review aimed to synthesize evidence on the differential impacts of extreme weather events on vector-borne disease transmission between urban and rural environments and identify settlement-specific prevention and healthcare preparedness strategies. **Methods**: A scoping review following PRISMA-ScR guidelines searched PubMed, EMBASE, Web of Science, and Scopus databases for studies examining climate–vector-borne disease relationships across settlement types. Sixteen empirical studies were analyzed using narrative synthesis, with urban–rural comparisons largely inferential given limited direct comparative studies. **Results**: From 6493 records identified, 4875 were screened after duplicate removal, yielding 16 studies for analysis. Studies covered multiple vector-borne diseases, including malaria, dengue, leishmaniasis, chikungunya, and Zika, across diverse geographic regions. Urban environments demonstrated infrastructure-mediated transmission dynamics characterized by heat island amplification exceeding vector survival thresholds, drainage system vulnerabilities creating breeding habitats, and density-driven epidemic spread affecting healthcare surge capacity. Rural settings exhibited ecosystem-mediated pathways involving diverse vector communities, agricultural breeding sites, and seasonal spillover from wildlife reservoirs, with healthcare accessibility challenges during extreme weather events. Critical research gaps included a limited number of longitudinal comparative studies and geographic variations in evidence generation. **Conclusions**: Extreme weather events create fundamentally distinct vector-borne disease transmission pathways across urban–rural gradients, necessitating settlement-specific prevention strategies and healthcare preparedness approaches. Evidence-based recommendations include urban infrastructure improvements, rural early warning systems, and cross-sectoral coordination frameworks to enhance the adaptive capacity for climate-resilient vector-borne disease prevention.

## 1. Introduction

Climate change has intensified extreme weather events all over the world, including heatwaves, droughts, floods, and tropical storms, which directly influence vector-borne disease (VBD) transmission through altered temperature, precipitation, and humidity patterns that affect vector survival and pathogen development [1,2]. VBDs, including malaria, dengue, chikungunya, Zika, and leishmaniasis, represent more than 17% of infectious diseases globally and cause over 700,000 annual deaths [3]. These diseases are mainly climate-sensitive due to their dependence on arthropod vectors, whose ecology and distribution are closely tied to specific environmental conditions [2]. With 3.6 billion people living in climate-vulnerable areas and an estimated 250,000 additional climate-related deaths projected annually by 2030–2050, VBDs pose escalating public health challenges [1]. Climate change functions as a threat multiplier for health systems, creating significant challenges for disease surveillance and control programs, particularly in resource-constrained settings where VBD transmission is endemic and adaptive capacity remains limited [1,4,5].

Recent surveillance data demonstrate concerning trends in VBD incidence that reflect climate change impacts on public health across settlement types. Global dengue transmission has expanded dramatically, with *Aedes* (*Ae.*) *aegypti* and *Ae. albopictus* mosquitoes now exhibiting distributions spanning all continents [6], creating expanding transmission potential particularly in urban environments where heat island effects maintain year-round breeding conditions [7,8]. Malaria showed initial declines globally from 864,000 deaths in 2000 to 576,000 in 2019, but recent increases have occurred, with 249 million cases reported in 2022 compared to 231 million in 2015 [9], partly attributed to climate-driven vector range expansion enabling *Anopheles* establishment at higher altitudes in East African and Central American highlands, and extreme weather disruptions to control programs [8,10]. Leishmaniasis cases demonstrate northward expansion in Europe and North America, with climate warming enabling *Phlebotomine* sandfly establishment in previously unsuitable regions, contributing to 0.7–1.2 million annual cutaneous leishmaniasis cases globally [11]. These epidemiological trends underscore the urgent need for settlement-specific climate-adapted VBD prevention strategies that address distinct infrastructure-mediated transmission pathways in urban areas and ecosystem-mediated pathways in rural settings.

Rapid urbanization has concentrated over half the world’s population in urban areas, creating distinct climate vulnerabilities across settlement types [12]. Urban and rural environments face contrasting climate risks and adaptive capacities, necessitating context-specific adaptation strategies [7,8]. These differential vulnerabilities require tailored approaches. Such approaches must address specific exposure patterns, susceptibility factors, and adaptive capacities across settlement types [13].

**Figure 1:**
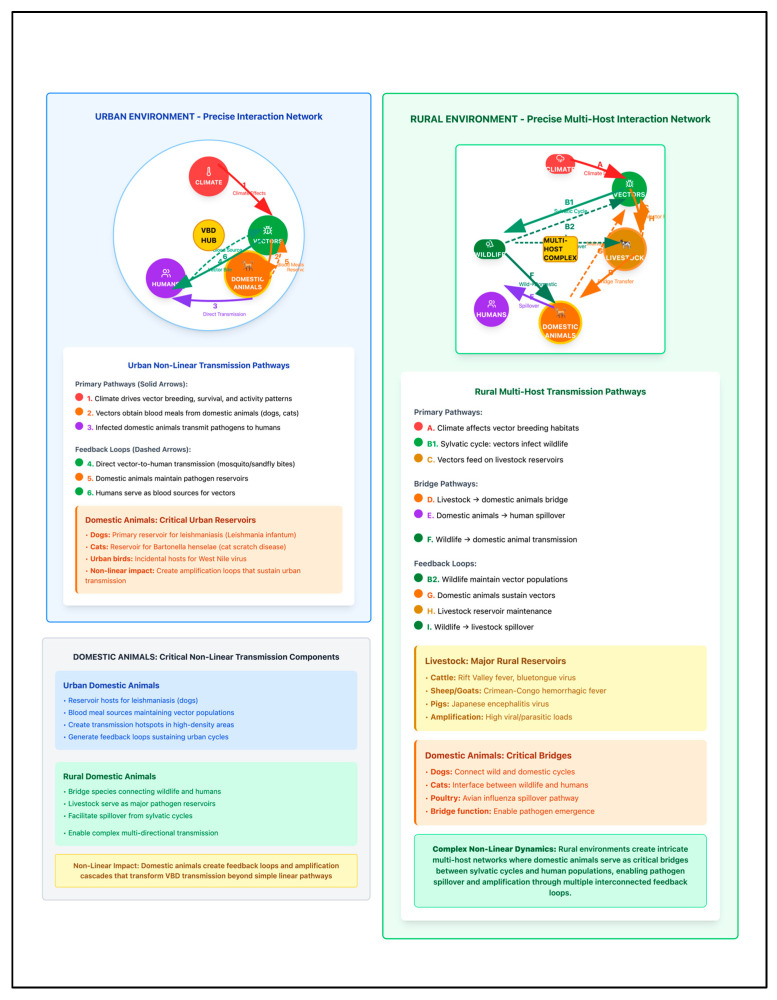
Comparative framework of vector-borne disease transmission pathways across urban (**left**) vs. rural (**right**) environments. Left panel: Urban infrastructure-mediated transmission featuring heat island effects, drainage vulnerabilities, and density-driven amplification. Right panel: Rural ecosystem-mediated transmission involving diverse vector communities, agricultural breeding sites, and wildlife spillover dynamics.

Temperature extremes fundamentally alter VBD transmission by modifying vector development rates and pathogen replication cycles. Warming temperatures accelerate mosquito development, while vector transmission shows distinct temperature-dependent optimization patterns for different *Aedes* species [14]. Viral replication demonstrates species-specific thermal optima [8]. Beyond these optimal ranges, extreme heat reduces vector survival and reproductive success [8,14]. Precipitation variability creates dynamic breeding habitat conditions for *Aedes* species through temporal lag effects in urban and peri-urban environments [15]. Heavy rainfall expands larval development sites while droughts concentrate vectors around remaining water sources. However, though temporal relationships vary by species and ecosystem characteristics [15]. Compound extreme events generate cascade effects that amplify transmission risk through synergistic impacts on vector ecology and human vulnerability patterns, with climate–health relationships exhibiting substantial regional variation driven by baseline climatic conditions, vector species composition, and local ecological contexts [16]. To illustrate these complex interactions and the fundamental differences between settlement types, a framework was developed that demonstrates how identical climate drivers create distinct transmission pathways through infrastructure-mediated versus ecosystem-mediated mechanisms across urban and rural environments (Figure 1). Understanding these mechanistic pathways is essential for developing early warning systems and targeted interventions that account for the complex, non-linear relationships between extreme weather events and VBD transmission dynamics across diverse geographic and climatic contexts [17] (Figure 1).

Building on the conceptual framework illustrated above, current research demonstrates that *Ae. aegypti* and *Ae. albopictus* mosquitoes now exhibit global distributions spanning all continents, creating expanding transmission potential for dengue, chikungunya, and Zika viruses [6]. Despite extensive research on climate–VBD relationships, comparative evidence examining differential impacts across urban versus rural settings remains critically limited. The framework highlights how settlement-specific mediating factors create fundamentally different epidemiological risks from identical climate drivers, yet most studies focus on single contexts without systematic comparison of transmission dynamics or intervention effectiveness between settlement types [18,19]. Most studies focus on single contexts without systematic comparison of transmission dynamics or intervention effectiveness between settlement types [8,16]. While mechanistic models demonstrate distinct thermal optima for different vector species and climate-driven mosquito density variations, their applications fail to examine how these relationships vary across urban–rural microenvironments with fundamentally different vector ecology conditions [14,15]. This evidence gap limits development of context-specific frameworks and targeted interventions that account for specific exposure patterns across settlement types. Comprehensive evidence synthesis is essential to inform climate adaptation planning and resource allocation [2].

This scoping review addresses the heterogeneous climate–VBD evidence by accommodating varied study designs and outcomes across diverse contexts. This review examines differential impacts of extreme weather events on VBD transmission across urban versus rural settings to inform evidence-based climate adaptation planning by identifying knowledge clusters and research gaps for targeted VBD prevention across settlement types [4]. The review provides a foundational framework for developing context-specific early warning systems and intervention strategies accounting for distinct urban–rural transmission dynamics [17].

## 2. Methodology

### 2.1. Study Design and Research Questions

**Figure 2:**
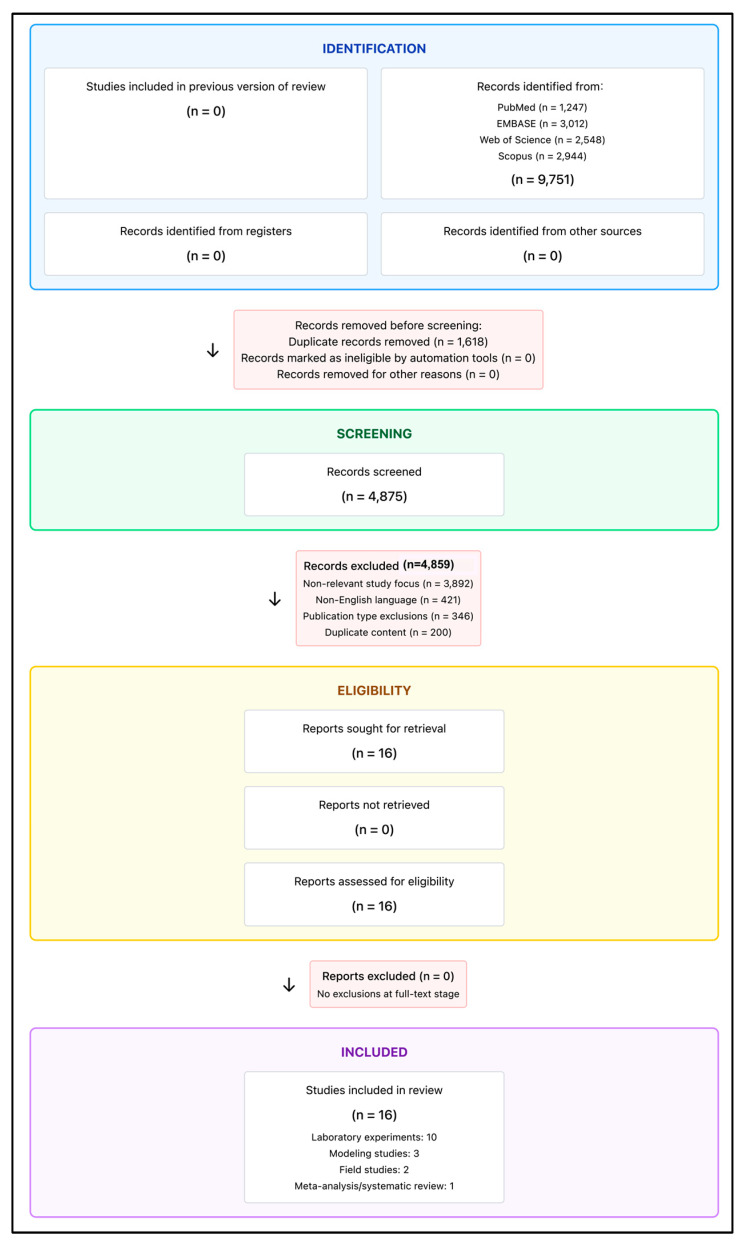
PRISMA flow diagram showing study selection process for scoping review of extreme weather impacts on vector-borne disease transmission across urban and rural settings. Source: Page MJ, et al. BMJ 2021;372:n71. doi: 10.1136/bmj.n71 [24].

This scoping review followed Preferred Reporting Items for Systematic Reviews and Meta-Analyses extension for Scoping Reviews (PRISMA-ScR) guidelines to systematically synthesize evidence on differential impacts of extreme weather events on VBD transmission across urban and rural settings [20]. The protocol for this scoping review was registered with INPLASY (International Platform of Registered Systematic Review and Meta-analysis Protocols) on 1 August 2025 (Registration No: INPLASY202580003). The methodology was selected to address broad, exploratory research questions and accommodate heterogeneous evidence spanning diverse study designs and contexts, making it suited for mapping the breadth and nature of research activity and identifying knowledge gaps [21] (Figure 2). The primary question, formulated using the Population–Concept–Context framework, was as follows: “What is known about how extreme weather events differentially affect VBD transmission dynamics in urban versus rural settings, and what evidence exists for adaptation strategies in each context?”. Secondary questions addressed mechanistic pathways, adaptation strategies, and knowledge gaps limiting understanding of urban–rural differences. The six-step methodological framework included (1) identifying research questions, (2) identifying relevant studies, (3) study selection, (4) data charting and extraction, (5) data synthesis and analysis, and (6) results reporting. The temporal scope covered 2000–2025 with literature searches conducted through 28 July 2025 and global geographic scope. The conceptual framework illustrating differential climate–VBD transmission pathways across urban and rural settings was developed based on systematic evidence synthesis and is presented as Figure 1 to guide the scoping review analysis. This conceptual framework advances existing climate–health models by explicitly distinguishing settlement-specific transmission pathways (infrastructure-mediated vs. ecosystem-mediated) and integrating domestic animal reservoirs across urban–rural gradients. This model demonstrates how identical climate drivers create fundamentally different epidemiological risks through settlement-specific mediating factors.

### 2.2. Eligibility Criteria and Search Strategy

The systematic literature search conducted across four major databases used climate terms (“climate change,” “extreme weather,” “heatwave,” “drought,” “flood”), vector-borne disease terms (“malaria,” “dengue,” “Zika,” “vector-borne”), settlement terms (“urban,” “rural,” “city,” “village”), and transmission terms (“outbreak,” “epidemic,” “incidence”) combined with Boolean operators (AND/OR). Climate change was operationally defined to encompass both gradual long-term climatic shifts (such as temperature increases enabling altitudinal disease expansion) and extreme weather events (including heatwaves, droughts, floods, and storms), reflecting the full spectrum of climate impacts on vector-borne disease transmission [22,23].

Inclusion criteria included quantitative studies, laboratory experiments, mathematical models, or surveillance analyses examining climate–vector-borne disease relationships; temperature, precipitation, or extreme weather impacts on vector ecology and disease transmission; peer-reviewed English-language publications (2000–2025); and studies involving public health-relevant vector species across any geographic setting. Exclusion criteria included studies lacking climate/environmental variables, focusing solely on non-vector-borne diseases, conference abstracts or unpublished reports, duplicate publications, and non-English studies published before 2000.

Following automated and manual duplicate removal using EndNote 21 (Clarivate Analytics, Philadelphia, PA, USA), 4875 unique records remained for systematic screening. Title and abstract screening excluded 4859 records due to non-relevant study focus (n = 3892), non-English language (n = 421), publication type exclusions (n = 346), and duplicate content (n = 200). Full-text assessment of 16 articles resulted in no additional exclusions, with all 16 studies providing climate–VBD data included in the final analysis using predefined criteria documented through citation management systems [21].

### 2.3. Data Charting, Extraction, Synthesis and Analysis

A standardized data extraction form was developed through an iterative process incorporating established scoping review guidelines, pilot-tested on 5 included studies, incorporating variables across bibliographic details, study characteristics (design, setting, population), extreme weather event types, VBD outcomes, urban–rural comparisons, mechanistic pathways, and adaptation strategies, with iterative refinement based on pilot testing results and single extraction by the author for data charting consistency [21]. To address single-author limitations, the author applied predefined inclusion/exclusion criteria with explicit operational definitions, utilized standardized data extraction forms with clear variable definitions, conducted systematic pilot testing to refine protocols, maintained detailed documentation of screening decisions with rationales, and performed cross-validation through re-extraction of a subset of studies to ensure consistency and minimize interpretation bias. Study quality was assessed using study-type-specific criteria: the Newcastle-Ottawa [25], adapted experimental quality criteria for laboratory studies (n = 6), and model validation criteria for modeling studies (n = 4), as detailed in Supplementary Table S2.

The complete data extraction form is provided in Appendix S2. Substantial heterogeneity in study designs, outcome measures, and regional contexts was systematically addressed through methodological stratification (categorizing by design type, temporal scale, and analytical approach), thematic grouping of diverse outcome measures (transmission metrics, incidence rates, vector abundance), and geographic contextualization accounting for baseline climatic conditions and socioeconomic factors. Where quantitative synthesis was not feasible due to methodological diversity, structured narrative synthesis was employed with explicit discussion of how contextual variations influenced observed relationships. Numerical summary characterized study distribution by geographic region, disease type, and methodological approach, while narrative synthesis identified recurring patterns in transmission mechanisms and adaptation strategies using an iterative approach to capture key concepts and themes from the heterogeneous evidence base [20]. Results were organized according to the urban–rural dichotomy and extreme weather event categories, with tabular summaries and descriptive mapping employed to illustrate knowledge clusters, research gaps, and geographic distribution of studies to provide a comprehensive overview of the evidence landscape and identify areas for future research.

## 3. Results

### 3.1. Search Results and Study Characteristics

The search yielded 6493 total records: 973 from PubMed, 778 from EMBASE, 2351 from Web of Science, and 2391 from Scopus, with complete database search strategies and detailed query syntaxes provided in Appendix S1. Building on the methodological framework outlined above, this section characterizes the current evidence base to contextualize the scoping review of climate–VBD relationships across urban and rural settings. The evidence base examining extreme weather impacts on VBD transmission demonstrates the critical role of climate variables in shaping disease dynamics across diverse geographical settings. Temperature and precipitation patterns are fundamental drivers of VBD transmission, with extreme weather events significantly influencing outbreak patterns, as exemplified by drought-induced West Nile virus amplification in Florida [26]. The systematic search revealed that urban environments often provide optimal breeding habitats for anthropophilic vectors, while rural transmission patterns depend heavily on local socioeconomic and environmental conditions, creating differential vulnerability patterns that intersect with existing health inequalities [27]. However, direct comparative studies examining these urban–rural differences remain critically limited, as detailed in the following sections. Current methodological approaches encompass epidemiological surveillance, mathematical modeling, and empirical analyses, though varied outcome measures and exposure definitions across studies present ongoing challenges for comprehensive evidence synthesis in climate–health research [4,28]. This methodological heterogeneity particularly complicates efforts to compare transmission dynamics across settlement types, necessitating the scoping review approach employed in this study to systematically map the breadth of evidence and identify knowledge gaps across diverse contexts.

### 3.2. Quality Assessment

Quality assessment using adapted quality criteria revealed variable study quality across the 16 included studies, with detailed individual ratings provided in Supplementary Table S2. Most studies demonstrated adequate methodology and data quality, though comparability between studies was limited due to methodological heterogeneity in exposure definitions and outcome measures.

The included studies demonstrated quantitative evidence on climate–vector-borne disease relationships (Table 1). These studies encompassed temperature and vector dynamics (4 studies), extreme weather events and disease outbreaks (4 studies), geographic range expansion and altitude shifts (3 studies), disease burden and geographic distribution (2 studies), and vector ecology and environmental factors (3 studies).

### 3.3. Climate Change and Extreme Weather Impacts on VBD Transmission

Heatwaves, operationally defined in this review as sustained periods of elevated temperatures exceeding local climatological norms that create thermal stress conditions for vector populations, exert complex, non-linear effects on VBD transmission through simultaneous impacts on vector physiology and pathogen development, with optimal transmission temperatures varying significantly among vector species and pathogen types [38]. Temperature–vector relationships vary significantly across species (Table 2), creating differential climate vulnerabilities that manifest distinctly across urban–rural gradients [29,30,35,36]. Non-mosquito vectors exhibit different tolerances, from narrow windows (*Glossina tsetse*: 22–26 °C) to cold survival capabilities (*Ixodes ticks*: inactive <7 °C) [29,35,36,39,40]. These species-specific thresholds (Table 2) create differential vulnerability patterns across urban–rural gradients where microclimate variations determine vector establishment success.

Regional manifestations of these temperature–vector relationships demonstrate global heterogeneity: Jakarta’s 4–6 °C heat island effects enable year-round Aedes transmission with projected dengue increases under 2 °C warming scenarios [7], East African highlands experience malaria expansion above 2000 m elevation through temperature-driven vector establishment [23], and Sahelian communities face drought-concentrated sandfly transmission contributing to 0.7–1.2 million annual leishmaniasis cases globally, with Mediterranean regions showing periurban *Phlebotomus* adaptation to warming microclimates [11].

Elevated temperatures accelerate mosquito development but reduce adult longevity, with critical thresholds and optimal ranges summarized in Table 2 [30]. Detailed physiological mechanisms are provided in Supplementary Materials. Critical survival thresholds demonstrate species-specific vulnerabilities: *Anopheles gambiae* experiences complete reproductive failure above 32 °C, larval mortality at 38 °C, and egg hatching failure at 40 °C [30]. Climate warming of 2–3 °C enables permanent vector establishment at higher altitudes, particularly affecting malaria transmission zones in highland regions of East Africa and Central America where temperature constraints historically limited *Anopheles* populations [10]. Conversely, extreme heatwaves exceeding 35 °C cause immediate reproductive failure in *Aedes* populations, while temperatures above 40 °C result in complete *Anopheles* population collapses, creating temporary but acute disruptions to transmission cycles [31].

Critically, long-term climate change establishes shifting baseline conditions enabling gradual vector range expansions and seasonal extensions, while extreme weather events trigger acute epidemic surges or temporary population collapses. Climate warming of 2–3 °C enables permanent *Anopheles* establishment at higher altitudes, whereas heatwaves exceeding 35 °C cause immediate reproductive failure in *Aedes* populations, while temperatures above 40 °C result in complete *Anopheles* population collapses, distinguishing chronic adaptation from acute disruption patterns [10,31].

West Nile virus transmission through *Culex* mosquitoes demonstrates specific temperature dependencies that exemplify the complex climate–vector–pathogen interactions across urban–rural gradients. Recent comprehensive analyses reveal that optimal WNV transmission occurs around 24 °C across *Culex* species, with species-specific development ranges spanning 16–35 °C and critical upper thresholds varying between 32–38 °C depending on the species [41]. These findings highlight the importance of species-specific approaches to climate risk assessment, as different *Culex* species exhibit varying thermal tolerances within the same geographic regions. Urban environments with heat island effects may push temperatures beyond optimal ranges for WNV transmission, while rural areas with lower baseline temperatures may experience enhanced transmission as warming approaches optimal development ranges. This temperature specificity for WNV transmission underscores the need for settlement-specific surveillance and control strategies that account for differential thermal environments across urban–rural gradients.

These fundamental temperature–vector relationships create the foundation for understanding differential impacts across settlement types, with urban heat island effects and rural baseline temperature variations modifying these core physiological responses in distinct ways, as examined in Section 3.4 and Section 3.5, respectively.

Precipitation extremes create contrasting yet equally disruptive impacts on VBD transmission through dynamic alterations in vector breeding habitats and human exposure patterns. Heavy rainfall and flooding events initially destroy existing breeding sites through high-velocity water flows but subsequently create extensive new larval development sites in stagnant pools, flooded basements, and temporary water containers [43]. These precipitation-driven breeding habitat changes manifest differently across urban and rural environments, with settlement-specific infrastructure and water management systems creating distinct vulnerability patterns detailed in Section 3.4 and Section 3.5. Dengue transmission shows significant increases following flood events due to expanded *Aedes* breeding opportunities particularly in urban areas where drainage infrastructure becomes overwhelmed, as further analyzed in Section 3.4 [28,44]. Conversely, drought conditions concentrate vector populations around diminishing water sources while simultaneously forcing communities to increase water storage in household containers, creating optimal breeding conditions for container-breeding vectors like *Ae. aegypti* [43]. Drought-induced amplification has been demonstrated with West Nile virus in Florida, where reduced water levels concentrated both vectors and avian hosts around remaining water bodies [26]. Infrastructure failures during extreme precipitation events disrupt vector control programs, compromise surveillance systems, and limit access to healthcare services [44]. Population displacement from flood-affected areas can introduce naive populations to endemic transmission zones or transport infected individuals to previously unaffected regions, creating spatially heterogeneous outbreak patterns [26,45]. Climate-induced population movements can spread VBDs to new geographic areas, with environmental migrants potentially carrying pathogens across epidemiological boundaries and overwhelming healthcare systems in receiving areas [44]. Floods also trigger mass hatching of flood-adapted species like *Ae. vexans* and *Culex tarsalis*, whose dormant eggs require inundation to activate, creating sudden vector population surges with up to 18-fold increases in larval densities within 2–3 weeks post-flooding [34,37].

Compound and sequential extreme weather events under climate change create synergistic impacts on VBD transmission that exceed the sum of individual hazards through complex multi-stressor interactions. Concurrent events such as heatwaves with droughts or flooding with temperature extremes generate amplified transmission risks by simultaneously expanding breeding habitats and accelerating vector development cycles [46]. Sequential extreme events trigger cascading system failures where initial infrastructure damage increases vulnerability to subsequent hazards, prolonging disease transmission windows and overwhelming vector control capacity [47]. Cumulative vulnerability assessments demonstrate that repeated extreme events deplete community resilience through deteriorating infrastructure, reduced economic resources, and weakened institutional capacity for disease prevention [43,48]. Recovery period dynamics critically determine long-term transmission patterns, as incomplete infrastructure restoration and reduced adaptive capacity between events create persistent transmission hotspots and may establish new endemic patterns in previously unaffected areas [43]. The temporal clustering of extreme events reduces ecological and social system recovery intervals, potentially overwhelming both vector population dynamics and human community adaptive capacity [49]. For example, Hurricane Katrina (2005) triggered a >2-fold increase in West Nile neuroinvasive disease incidence in hurricane-affected Louisiana and Mississippi regions during 2006, demonstrating how infrastructure damage and novel breeding habitat creation can sustain transmission long after the initial extreme weather event [32].

### 3.4. Urban Settings: Infrastructure-Mediated Transmission Dynamics and Vulnerabilities

Urban infrastructure creates distinct climate vulnerabilities through cascading failures that fundamentally alter VBD transmission patterns. During extreme weather events, drainage system overwhelm transforms urban landscapes into extensive vector breeding habitats, while inadequate stormwater management creates persistent water pooling optimal for container-breeding vectors [28,50]. Urban green spaces provide habitat for multiple vector types including ticks in vegetated areas and sandflies in sheltered microclimates [51]. Building design in densely populated areas facilitates vector access through inadequate screening and vertical transmission pathways in multi-story buildings [6,43]. Urban animal populations serve as reservoir hosts for multiple vector-borne pathogens, creating transmission cycles involving ticks, sandflies, and mosquitoes [52]. Transportation disruptions during extreme weather limit healthcare access, disrupt vector control, and create displacement patterns that introduce naive populations to endemic zones [45,47]. High population density amplifies transmission through increased human–vector contact rates, creating conditions for rapid epidemic spread when vector populations establish near large susceptible population [15,53]. Healthcare systems face critical surge capacity limitations during climate-induced outbreaks, while social distancing challenges in crowded environments reduce non-pharmaceutical intervention effectiveness [4,43,48].

Urban–rural transmission dynamics interconnect through human mobility and vector dispersal networks. Urban–rural transmission interconnects through daily commuting transporting infected individuals, commercial transport facilitating vector introductions (*Ae. albopictus* global expansion via tire shipments), and animal movement facilitating tick-borne disease and leishmaniasis transmission between zones [44,54]. These climate-driven migrations often result in settlement within urban slums lacking adequate sanitation and healthcare infrastructure, facilitating rapid disease transmission among vulnerable displaced populations [44].

Urban heat islands amplify temperatures by 2–8 °C above rural baselines [55]. Infrastructure vulnerability patterns are described in Appendix S2. Urban green spaces create diverse vector habitats, with vegetated areas and ecological corridors facilitating tick establishment and supporting various arthropod vectors [56]. Urban trees and vegetation provide important cooling effects, reducing temperatures by 1–4 °C, offering critical mitigation potential within the thermal constraints outlined in previous sections. Air conditioning access disparities create differential exposure risks, affluent neighborhoods experience reduced indoor vector exposure while disadvantaged communities face compounded vulnerabilities from increased vector exposure and heat-related health impacts that compromise immune function [14,27,57]. These thermal heterogeneities create persistent transmission foci where optimal breeding conditions coincide with vulnerable populations, with lower-income neighborhoods often experiencing more severe heat exposure and reduced adaptive capacity compared to higher-income areas, necessitating fine-scale spatial vector control approaches [16,30,58].

### 3.5. Rural Settings: Ecosystem-Mediated Transmission Dynamics and Vulnerabilities

Applying the temperature-driven vector development principles outlined in previous sections to rural contexts. Both rural and urban areas face unique climate-driven VBD challenges due to their proximity to diverse ecosystems, agricultural ecosystems and natural habitats in rural areas, and modified urban ecosystems in cities, that support diverse vector populations [59,60]. Climate change is fundamentally reshaping vector ecology through altered temperature and precipitation patterns that modify breeding habitats and development cycles [60].

Sandfly vectors utilize agricultural structures and animal shelters for breeding and resting sites [61]. Climate-induced changes in crop patterns alter the distribution of disease reservoirs and vector species [18,19]. In contrast to urban heat island amplification, rural areas with lower baseline temperatures may experience enhanced transmission as warming approaches optimal development ranges, with rural communities experiencing direct exposure to sylvatic transmission cycles as deforestation and agricultural expansion bring human populations into contact with wildlife disease reservoirs [2,38,59]. Specifically, temperature increases of 2–3 °C in rural East Africa and Central America highland areas can enable mosquito establishment at previously unsuitable altitudes, expanding malaria transmission zones [10,14,18]. Similarly, warming expands tick habitat ranges into higher elevations, introducing tick-borne diseases to previously unaffected rural populations. Unlike urban environments with year-round breeding conditions created by heat islands, rural areas also experience greater seasonal variation in vector abundance due to dependence on natural water sources and agricultural cycles, creating distinct transmission windows that require specialized surveillance and control strategies [15,43,59]. Evidence for rural transmission dynamics relies on less studies (n = 4) compared to urban findings (n = 6), indicating rural conclusions require more cautious interpretation pending additional research.

Climate extremes disrupt vertebrate reservoir dynamics in rural agricultural settings through cascading ecological effects. Drought concentrates mosquito and wildlife hosts around limited water sources while inducing stress-related immunosuppression [62,63]. Tick vectors remain quiescent in vegetation during drought [64], while sandflies concentrate around livestock shelters [65]. Mosquito feeding behavior adapts dynamically to host availability, *Culex* mosquitoes switch from declining avian to mammalian hosts during extreme weather [66,67]. Flooding disperses infected populations while creating new breeding habitats [68,69].

While urban centers face significant water challenges, rural areas experience distinct but equally severe climate-induced water vulnerabilities that compromise healthcare delivery. Climate change profoundly disrupts global water systems, directly threatening both human health and healthcare infrastructure. Rising temperatures and altered precipitation patterns are reducing water availability in critical regions, with 1.4 billion people already living in water-stressed regions and rural communities often lacking redundant water sources, healthcare systems face cascading failures from compromised water quality and extreme weather [57]. In rural settings, where healthcare facilities are already limited and often located hours from affected communities, flooding events trigger disease outbreaks, exemplified by West Nile virus outbreaks following Hurricane Katrina in Louisiana and Mississippi [32]. Rural agricultural systems create complex vector-pathogen dynamics, with extensive agriculture reducing VBD transmission through pesticide use and land cultivation that decreases vector abundance, while intensive rice agriculture increases mosquito breeding opportunities and disease risk [59]. Rural water management challenges are compounded by agricultural demands, with irrigation and livestock needs competing with domestic water supplies during drought periods, forcing communities to rely on potentially contaminated surface water sources that facilitate vector breeding [27,43]. Rural areas particularly benefit from nature-based solutions such as wetland restoration and watershed management, which offer critical adaptation through flood protection, temperature regulation, and water quality improvement while supporting local livelihoods through ecosystem services [13,70]. However, successful implementation requires coordinated governance frameworks that integrate climate adaptation with health system strengthening, particularly in vulnerable rural regions where inadequate infrastructure amplifies climate risks and limited transportation networks impede emergency response during extreme weather events [43,57].

### 3.6. Comparative Analysis: Urban vs. Rural Differences

Infrastructure-mediated transmission in urban settings and ecosystem-mediated transmission in rural settings create fundamentally different vector ecology, human exposure, and pathogen dynamics, as illustrated in Figure 1. Urban environments support concentrated populations of container-breeding vectors like *Ae. aegypti* in artificial breeding sites, creating high-density transmission foci where population concentration amplifies human–*Aedes* contact rates and enables rapid epidemic spread through *Aedes*-human cycling [6,15]. Infrastructure-mediated transmission pathways in urban areas are characterized by drainage system failures, building design vulnerabilities, and transportation disruptions that create cascading effects during extreme weather events. Conversely, rural areas maintain diverse vector communities including sylvatic species that facilitate complex enzootic-epizootic transmission cycles, where agricultural activities and proximity to natural habitats expose populations to zoonotic spillover risks during specific occupational periods [16,59]. Rural transmission dynamics involve ecosystem-mediated pathways where wildlife reservoir circulation creates seasonal spillover events dependent on agricultural and ecological factors, contrasting sharply with sustained human–vector cycles observed in urban settings [18,53]. Pathogen circulation patterns reflect these ecological differences, with urban transmission characterized by sustained human–vector cycles among naive populations and peridomestic wildlife reservoirs (birds, rodents) that facilitate enzootic maintenance, while rural dynamics involve wildlife reservoir circulation that creates seasonal spillover events dependent on agricultural and ecological factors [18,53]. Seasonal transmission timing varies markedly across settlement types, as urban heat islands eliminate distinct seasonality by maintaining year-round breeding conditions and extended vector activity, whereas rural transmission follows pronounced seasonal windows driven by agricultural cycles, precipitation-dependent breeding sites, and temperature fluctuations that necessitate temporally targeted intervention strategies [8,28].

Socioeconomic vulnerability patterns differ markedly between urban and rural settings: urban vulnerability operates through density-driven amplification and income-based spatial segregation, while rural vulnerability manifests through structural disadvantages compounding ecosystem-mediated transmission during extreme weather events [27,43,57]. Infrastructure resilience exhibits contrasting patterns, as urban systems provide higher baseline capacity but experience greater complexity-induced failure cascades during extreme events, while rural infrastructure demonstrates lower overall capacity yet different failure modes potentially addressable through localized interventions [50,57]. Healthcare capacity disparities reflect these vulnerabilities, with urban centers offering concentrated medical resources but facing surge capacity limitations during climate-induced outbreaks, contrasting sharply with rural areas where baseline access challenges are severely exacerbated by extreme weather disruptions to transportation and communication networks [43,53]. Community preparedness levels vary fundamentally, as urban populations access formal early warning systems despite limited environmental awareness, while rural communities possess traditional ecological knowledge and adaptation practices but face institutional capacity constraints that limit coordinated outbreak response and vector control implementation [48,59,71].

### 3.7. Adaptation and Response Strategies

Figure 1 shows that effective adaptation strategies must address distinct transmission pathways and mediating factors across settlement types. Urban interventions must target infrastructure-mediated transmission through drainage system improvements, building design modifications, and surge capacity development while leveraging technological capacity and concentrated resources. Rural strategies must focus on ecosystem-mediated transmission through agricultural modifications, natural resource management, and community-based approaches that integrate traditional ecological knowledge with scientific interventions. Cross-cutting strategies require integrated early warning systems that account for differential climate sensitivities and compound event impacts illustrated in the framework.

Urban interventions rely on limited empirical evidence from Hurricane Katrina’s West Nile virus impacts [23] and Florida drought amplification [25]. Infrastructure and technology recommendations are largely extrapolated from general climate–health literature rather than consistent findings across reviewed studies. Urban adaptation strategies leverage infrastructure improvements and technological capacity to address climate-driven VBD risks in high-density environments [72]. Infrastructure resilience improvements include climate-proofing drainage systems, implementing sustainable urban drainage systems, and adopting flood-resistant building designs that minimize mosquito breeding sites while maintaining urban functionality [50].

Tick management in urban areas requires vegetation management in parks and green spaces [73], while sandfly control focuses on reducing organic matter accumulation and improving sanitation in livestock-adjacent areas [74]. Urban planning modifications integrate vector control into zoning regulations and building codes, while strategic green space distribution provides cooling benefits without creating mosquito habitats but requires careful vegetation selection to minimize tick habitat [70]. Technology-based surveillance systems utilize remote sensing, geographic information systems, and mobile health platforms for rapid outbreak detection and targeted interventions, while community engagement leverages social networks through neighborhood-based programs and participatory approaches for sustained vector control activities [53].

Rural adaptation emphasizes ecosystem-based solutions aligned with agricultural livelihoods and traditional knowledge systems that integrate indigenous knowledge with scientific approaches to develop culturally relevant and effective climate adaptation strategies [75]. Agricultural modifications include integrated pest management, improved irrigation practices, and livestock management incorporating zooprophylaxis to reduce mosquito-human contact [59]. Tick control in agricultural settings emphasizes pasture rotation, wildlife exclusion, and targeted acaricide applications [76], while sandfly management focuses on livestock shelter modifications and organic waste management [74]. Natural resource management integrates watershed restoration and biodiversity conservation as nature-based solutions providing flood protection and vector habitat modification [13]. Community health worker programs provide essential decentralized surveillance and case management in areas with limited healthcare access [71]. Cross-cutting strategies require integrated early warning systems combining meteorological, entomological, and epidemiological data for proactive interventions [17], while health system strengthening emphasizes surge capacity development and cross-sectoral coordination mechanisms linking health, environment, and planning sectors to address climate-driven disease risks through coordinated governance frameworks [4]. Rural evidence was severely limited, with only altitude-based malaria interventions [23] and agricultural vector management documented [59]. Community health worker and ecosystem-based recommendations represent broader adaptation principles lacking direct empirical support in our reviewed literature.

## 4. Evidence Gaps and Research Needs

Analysis of the 16 studies reveals critical knowledge gaps that impede comprehensive understanding of climate–VBD relationships across settlement types (Table 1). Most significantly, only three studies explicitly examined urban–rural differences, with the majority focusing on single contexts without systematic comparison of transmission dynamics or intervention effectiveness between settlement types. The framework highlights how compound and sequential extreme events create synergistic impacts that exceed individual hazard effects, yet current research predominantly examines single climate variables rather than complex multi-stressor interactions. Furthermore, the mediating role of infrastructure and socioeconomic factors illustrated in the framework remains inadequately quantified, particularly regarding how these factors modify climate–vector–health relationships across different settlement contexts. Quality assessment using the Newcastle-Ottawa Scale [25] revealed variable study quality across the 16 included studies, with detailed ratings provided in Supplementary Table S2.

The included studies demonstrate pronounced geographic inequities, with six studies from North America and only four from Africa, despite Sub-Saharan Africa bearing the highest VBD burden. Specifically, the geographic distribution included studies from the United States (six studies), Ghana, Kenya, Côte d’Ivoire, and Ethiopia (four African studies), China (one study), Colombia (one study), Latin America regional analysis (one study), Greece (one study), Sweden (one study), and global analyses (one study). Geographic inequities are pronounced, with six studies from North America versus four from Africa, substantially overrepresenting high-income temperate regions while underrepresenting tropical areas of Sub-Saharan Africa, Southeast Asia, and rural Latin America where VBD burden is highest [18,53,59]. Temporal research gaps are particularly evident, as ten of the 16 studies employed cross-sectional or short-term designs rather than longitudinal analyses needed to establish causal relationships, with long-term follow-up studies spanning multiple transmission seasons critically lacking and seasonal variation documentation remaining incomplete across endemic regions [15,17,26,28,38,43]. Methodological heterogeneity across the 16 studies severely impedes evidence synthesis, with diverse outcome measures ranging from vector abundance to disease incidence, inconsistent climate exposure definitions, and varying temporal scales preventing robust comparative analysis. Standardized impact measurement frameworks and comparative study designs explicitly contrasting urban versus rural transmission dynamics remain exceptionally rare [4,6,17,28,53,59]. Data quality limitations are evident in eight of the 16 studies, particularly those from resource-limited settings where surveillance capacity is inadequate, vector monitoring is sporadic, and meteorological data coverage is incomplete [43,71]. Furthermore, only four of the 16 studies employed mathematical modeling approaches, and these often relied on simplified climate-disease relationships that fail to capture complex interactions between environmental variables, socioeconomic factors, and vector ecology, while climate scenario-specific evidence remains severely limited with most projections based on general rather than extreme weather-specific impacts [8,16,19,38,50]. The limited empirical evidence base (n = 16) compared to the broader literature (total references) highlights the critical scarcity of high-quality climate–VBD research and creates substantial uncertainty in climate–health projections limiting the development of evidence-based adaptation strategies tailored to specific geographic and climatic contexts.

## 5. Discussion

### 5.1. Key Findings: Differential Climate–VBD Dynamics Across Settlement Types

This analysis reveals that current vector-borne disease surveillance and control strategies did not succeed in accounting for fundamentally different climate-mediated transmission mechanisms across urban versus rural environments—a critical oversight with immediate public health implications. These mobility-driven interconnections detailed in Section 3.4 create transmission bridges between settlement types through human migration and vector dispersal networks, contrasting with the distinct ecological patterns within each setting. Heat island effects create urban–rural temperature differentials that modify vector survival patterns differently across settlement types, with implications detailed in Section 3.4 and Section 3.5 [30]. This contrasts with rural environments where lower baseline temperatures enable enhanced transmission as warming approaches optimal development ranges for vector development and pathogen replication [77]. Environmental changes including deforestation and water control projects significantly reshape VBD transmission patterns across settlement types, with replacement of natural habitats by agricultural systems creating new ecological niches that support different vector species and facilitate the conversion of vectors from primarily zoophyllic to anthrophyllic orientation [78]. These differential thermal and ecological impacts, combined with contrasting precipitation-driven breeding dynamics where urban drainage system overwhelm generates extensive artificial breeding sites during floods while rural communities face seasonal transmission windows linked to agricultural irrigation cycles and water storage dependencies [78,79], underscore the critical need for settlement-specific adaptation strategies. Given these contrasting vulnerabilities across settlement types, nature-based solutions offer important mitigation potential, with urban green infrastructure providing cooling effects of approximately 1 °C while rural watershed management addresses both flood protection and vector habitat modification [55]. These differential pathways identified through systematic evidence synthesis demonstrate that effective climate adaptation for VBD prevention requires fundamentally different approaches across urban and rural contexts, with direct implications for the resource allocation, intervention design, and early warning system development discussed in subsequent sections.

### 5.2. Vulnerability Patterns and Adaptive Capacity Analysis

Building on the transmission dynamics identified above, the scoping review reveals that urban and rural vulnerability to climate-driven VBDs operate through fundamentally contrasting mechanisms that amplify these differential pathways. Urban vulnerability emerges through density-driven amplification effects where high population concentrations facilitate rapid epidemic spread via increased human–vector contact rates, while income-based spatial segregation concentrates vulnerable populations in areas with inadequate housing and limited climate protection—precisely where heat island effects create optimal vector breeding conditions [27,57]. Despite higher baseline medical infrastructure, urban healthcare systems face critical surge capacity limitations during climate-induced outbreaks when concentrated populations overwhelm emergency services, particularly affecting disadvantaged communities residing in microenvironmental hotspots most favorable for vector proliferation [4,55]. Conversely, rural vulnerability manifests through structural disadvantages that compound ecosystem-mediated transmission, where baseline poverty and climate-dependent livelihoods create cascading vulnerabilities during extreme weather events that compromise already fragile healthcare, transportation, and water infrastructure systems, effectively isolating communities when VBD risks peak [43,71]. Differential exposure mechanisms reflect distinct risk profiles—rural populations face seasonal vector contact through agricultural activities and proximity to natural breeding habitats, contrasting with continuous urban exposure patterns—while adaptive capacity analysis reveals complementary but underutilized strengths: urban areas leverage technological advantages including early warning systems and coordinated emergency response, whereas rural communities possess traditional ecological knowledge and community-based adaptation practices that remain institutionally under-supported despite their local relevance [17,59,75]. These contrasting vulnerability patterns have important implications for intervention design and resource allocation, as explored in the following section.

### 5.3. Settlement-Specific Implementation Requirements and Policy Implications

These findings demand immediate reconsideration of climate–VBD preparedness frameworks. Current one-size-fits-all approaches ignore the distinct transmission ecologies identified here, potentially undermining intervention effectiveness as extreme weather intensifies. Urban areas face particular challenges from climate change including heat island effects that can extend vector breeding seasons and extreme weather events that disrupt infrastructure, requiring interventions that address density-driven disease transmission and climate-related infrastructure vulnerabilities [48,53]. Rural approaches must address limited healthcare access and infrastructure gaps that are exacerbated during extreme weather events, with community-based health systems providing essential surveillance and response capacity where formal healthcare systems are compromised [71].

Cross-cutting strategies emerge as critical for addressing the complex climate–health interactions identified in this review. These require integrated early warning systems combining meteorological, entomological, and epidemiological data to address compound extreme events, alongside health system strengthening that prioritizes surge capacity development and cross-sectoral coordination mechanisms [4,80]. Land-use intensification affects vector-borne disease transmission differently across urban and rural contexts, with medium land-use intensity associated with higher infection prevalence, while both low and high intensities show reduced transmission risks [59]. Multi-hazard early warning systems must address the complex interactions between climate extremes and disease risks, particularly given that populations in the Global South face multiple overlapping hazards simultaneously [80]. Health system strengthening must prioritize surge capacity development and preparedness for compound events, as extreme weather can overwhelm healthcare infrastructure while simultaneously increasing disease burden [4,48]. Sustainability mechanisms must integrate climate adaptation with health system resilience through governance frameworks that recognize the interconnected nature of climate and health risks [17]. Financing strategies must support context-appropriate approaches that account for the differential vulnerabilities between urban and rural populations, particularly focusing on protecting the most vulnerable communities who are disproportionately affected by both climate change and vector-borne diseases [50,81]. These recommendations align with WHO’s Global Vector Control Response framework for integrated vector management [82], the Sendai Framework for Disaster Risk Reduction’s emphasis on ecosystem-based adaptation [83], and SDG 3.4 targets for reducing non-communicable disease mortality through prevention and treatment [84].

### 5.4. Future Directions and Recommendations

Longitudinal comparative studies explicitly contrasting urban versus rural transmission dynamics represent the most urgent research need, requiring standardized methodological frameworks and multi-stressor interaction research examining compound extreme events through advanced modeling techniques that capture synergistic climate–health relationships [4,17,46,47]. Development of consolidated comparison matrices systematically contrasting urban versus rural transmission pathways, vulnerability patterns, and adaptation strategies represents a critical research priority that could reduce analytical redundancy and enhance policy-relevant evidence synthesis. Geographic research equity demands substantial investment in surveillance capacity across Sub-Saharan Africa, Southeast Asia, and rural Latin America where VBD burden is highest yet evidence remains critically limited, while mechanistic research must quantify how infrastructure and socioeconomic factors mediate climate–vector–health relationships across urban–rural gradients [18,50,53,59,78]. Integrated surveillance platforms combining meteorological, entomological, and epidemiological data should leverage remote sensing and mobile health technologies for real-time outbreak prediction, while participatory research approaches must systematically integrate traditional ecological knowledge with scientific monitoring, particularly in rural contexts where community-based adaptation practices remain under-utilized [17,53,59,75]. Health system strengthening must prioritize surge capacity development and multi-hazard preparedness through cross-sectoral coordination frameworks that integrate climate adaptation with health system resilience, supported by financing mechanisms addressing differential vulnerabilities and scaled implementation of nature-based solutions including urban green infrastructure and rural watershed management. Vector-specific surveillance and control strategies must address the distinct ecology of different vector types: tick monitoring in urban green spaces and rural pastures [73,76], sandfly control through livestock shelter modifications and organic waste management [74], and tsetse fly surveillance using satellite-based breeding site identification in agricultural areas [4,13,35,48,55,70,71,80,81]. Implementation pathways should prioritize international funding for comparative research building local capacity through South–South collaboration, early warning system development co-designed with affected communities incorporating settlement-specific transmission dynamics, and interdisciplinary capacity building programs strengthening institutional frameworks for climate–health research and practice across the urban–rural continuum, consistent with WHO’s Global Vector Control Response operational framework [82] and Sendai Framework implementation priorities [4,17,18,57,80,81,83]. These recommendations provide a roadmap for evidence-based climate adaptation that accounts for the differential transmission dynamics and vulnerability patterns documented across settlement types, moving beyond one-size-fits-all approaches toward targeted interventions that harness settlement-specific strengths while addressing distinct structural limitations.

### 5.5. Study Limitations

This study has several limitations that must be acknowledged when interpreting the findings. The limited number of empirical studies (n = 16) providing direct climate–VBD evidence, compared to the broader references cited, highlights the scarcity of high-quality empirical research in this field and may limit the generalizability of findings. Both the study selection and data extraction processes were conducted solely by the author. While the author acknowledges the use of pilot testing and standardized extraction forms, the absence of multiple reviewers introduces the risk of selection and interpretation bias. The single-author approach to study selection and data extraction, while mitigated through standardized protocols and pilot testing, may introduce selection bias compared to multi-reviewer approaches. Future research would benefit from incorporating multiple reviewers or adjudication mechanisms to further enhance methodological rigor and minimize potential bias in study inclusion decisions and data interpretation. The uneven distribution of empirical evidence (4 rural-focused versus 6 urban-focused studies) limits confidence in rural-specific conclusions compared to urban findings. Publication bias may favor urban-focused research from well-resourced institutions over rural studies from resource-limited settings, potentially overrepresenting urban evidence despite higher VBD burden in rural areas. The evidence was taxonomically constrained, with 12 of 16 studies focusing on mosquito-borne diseases, limiting generalizability to other vector systems. Language restrictions to English publications likely excluded relevant studies from non-English speaking regions where VBD burden is highest, potentially underrepresenting evidence from Sub-Saharan Africa, Latin America, and Southeast Asia, potentially biasing findings toward lower-burden English-speaking countries. The substantial methodological heterogeneity across the 16 studies, with inconsistent outcome measures, exposure definitions, and temporal scales, limited the ability to conduct robust comparative analysis and may have obscured important urban–rural differences in climate–VBD relationships. This heterogeneity was systematically managed through methodological stratification and structured narrative synthesis, though the fundamental diversity in approaches prevented quantitative meta-analysis and required interpretive frameworks that may introduce analytical limitations. Additionally, the rapid evolution of climate science and the publication lag inherent in academic literature mean that the study findings may not capture the most recent developments in climate–VBD modeling and extreme weather impacts, particularly regarding compound events and tipping point dynamics. Despite these limitations, this scoping review provides a comprehensive synthesis of comparative urban–rural climate–VBD evidence and establishes a foundation for future research addressing the critical knowledge gaps identified throughout this review.

## 6. Conclusions

Based on inferential analysis across predominantly single-setting studies, extreme weather events create fundamentally distinct climate-mediated transmission pathways for vector-borne diseases across urban and rural settings. Urban environments demonstrate infrastructure-mediated dynamics characterized by heat island amplification, drainage system vulnerabilities, and density-driven epidemic spread, while rural settings exhibit ecosystem-mediated pathways involving diverse vector communities, agricultural breeding habitats, and seasonal spillover events from wildlife reservoirs. Critical research gaps—particularly the scarcity of longitudinal comparative studies and geographic inequities in evidence generation—underscore the urgent need for standardized methodological frameworks and enhanced surveillance capacity in high-burden regions of Sub-Saharan Africa, Southeast Asia, and rural Latin America. The evidence-based recommendations provide a roadmap for climate-resilient vector-borne disease prevention that moves beyond one-size-fits-all approaches toward targeted interventions harnessing settlement-specific strengths while addressing distinct structural vulnerabilities, ultimately advancing adaptive capacity across the urban–rural continuum in an era of intensifying climate change.

## Figures and Tables

**Table 1:** Characteristics of Included Empirical Studies (n = 16).

No.	Study	Year	Geographic Location	Study Type	Climate Factor	Vector/ Disease	Key Findings
Temperature and Vector Dynamics							
**1.**	**Brady et al.** [29]	2014	Global	Modeling/Laboratory	Temperature	*Aedes* spp./Dengue	*Ae. aegypti*: 14–34 °C range; *Ae. albopictus*: 13–29.4 °C; 42× higher suitability for albopictus
**2.**	**Agyekum et al.** [30]	2022	Ghana	Laboratory	Elevated temperature	*Anopheles gambiae*/Malaria	Temperature effects on mosquito development and survival
**3.**	**Ciota et al.** [31]	2014	USA	Laboratory	Temperature	*Culex* spp./West Nile	Temperature-dependent life history trait variations
**4.**	**Tesla et al.** [8]	2018	Global	Empirical/Modeling	Temperature	Ae. spp./Zika	Optimal transmission at 29 °C; unimodal temperature response
**Extreme Weather Events and Disease Outbreaks**							
**5.**	**Caillouët et al.** [32]	2008	Louisiana, Mississippi, USA	Surveillance	Hurricane	West Nile virus	0→11 cases (Louisiana), 0→10 cases (Mississippi) post-Katrina; 2-fold increase in 2006
**6.**	**Shaman et al.** [26]	2005	Southern Florida, USA	Surveillance	Drought	West Nile virus	Drought amplification of WNV transmission
**7.**	**Danforth et al.** [33]	2016	California, USA	Laboratory/Modeling	Temperature cycling	West Nile virus	Impact of diurnal temperature variation on transmission
**8.**	**Mourelatos et al.** [34]	2025	Thessaly, Greece	Surveillance	Extreme flooding	West Nile virus	Flood-associated WNV transmission increase
**Geographic Range Expansion and Altitude Shifts**							
**9.**	**Siraj et al.** [23]	2014	Ethiopia, Colombia	Surveillance	Temperature increase	Malaria	200 m upward shift (Ethiopia), 180 m (Colombia); 2.3 °C temperature correlation
**10.**	**Colón-González et al.** [7]	2018	Latin America	Modeling	Temperature scenarios	Dengue	1.5–2 °C limit could reduce dengue incidence and spread
**11.**	**González et al.** [19]	2010	North America	Ecological modeling	Climate change	Leishmaniasis	Northward expansion predictions for vector and reservoir species
**Disease Burden and Geographic Distribution**							
**12.**	**Alvar et al.** [11]	2012	Global	Surveillance/Review	Climate factors	Leishmaniasis	0.2–0.4 M VL cases, 0.7–1.2 M CL cases annually; 90% VL in 6 countries
**13.**	**Li et al.** [15]	2019	China	Surveillance	Climate variation	Dengue	Mosquito density predicts spatiotemporal dengue dynamics
**Vector Ecology and Environmental Factors**							
**14.**	**Gachoki et al.** [35]	2021	Kenya	Satellite modeling	Environmental factors	Tsetse flies/Trypanosomiasis	Satellite-based breeding site identification
**15.**	**Konan et al.** [36]	2023	Côte d’Ivoire	Field study	Environmental factors	Tsetse flies/Trypanosomiasis	Urban forest ecology and transmission risk
**16.**	**Lindström et al.** [37]	2021	Sweden	Field study	Flooding	Floodwater mosquitoes	Species-specific hatching responses to flood environments

**Table 2:** Temperature Optima and Critical Thresholds for Vector-Borne Disease Transmission.

**Vector Species**	**Development Temperature Range (°C)**	**Critical Lower Threshold (°C)**	**Critical Upper Threshold (°C)**	**Pathogen**	**Optimal Transmission Temperature (°C)**	**Data Source**	**Reference**
***Ae. aegypti***	Immatures: 16–35 °C	~16 °C	>35 °C (reproductive failure), >40 °C (death point)	Zika virus	28.9 °C	Lab	(Tesla et al., 2018) [8]
***Ae. aegypti***	Variable by trait	Variable by trait	>35 °C	Dengue, Chikungunya	29.1 °C	Lab/Model	(Mordecai et al., 2020) [14]
***Ae. albopictus***	10.4–35 °C	10.4 °C	>35	Dengue, Chikungunya	26.4 °C	Lab/Model	(Mordecai et al., 2020) [14]
***Anopheles gambiae***	25–34 °C (study range)	Not specified	>32 °C (reproductive failure), >38 °C (larval mortality), >40 °C (egg hatching failure)	Malaria	25–28 °C	Lab	(Agyekum et al., 2022) [30]
***Phlebotomine sandflies***	Variable by species	Variable by species	>30	Leishmaniasis	20–30	Field review	(Ready 2010) [39]
***Ixodes* ticks**	Variable by species/stage	>7 °C (activity threshold)	Variable by species	Lyme disease	>7	Field/Model	(Randolph 2004) [40]
***Glossina* tsetse flies**	Not clearly specified	Not clearly specified	>32 °C (mortality risk)	Trypanosomiasis	22–26 °C	Lab/Field	(Konan et al., 2025; Gachoki et al., 2021) [35,36]
***Culex* mosquitoes**	Species-specific variation	Variable by species	Species-specific: 32–38 °C range	West Nile virus	~24 °C	Lab/Model	(Heidecke et al., 2025; Moser et al., 2023) [41,42]

**Note**: Many references focus on specific temperature ranges tested rather than comprehensive thresholds across all life stages. Where data are incomplete, this is noted rather than extrapolated.

## Data Availability

No new data were created or analyzed in this study. Data sharing is not applicable to this article.

## Supplementary Materials

The following supporting information can be downloaded at: https://www.mdpi.com/article/10.3390/healthcare13192425/s1, Appendix S1 provides complete database search strategies with specific query syntaxes, controlled vocabulary terms, and filter applications for PubMed, EMBASE, Web of Science, and Scopus. Table S1 presents the database search results and filtering outcomes showing initial retrieval numbers, records after filter application, and final inclusion counts across all four databases. Table S2 presents Newcastle-Ottawa Scale quality assessments for all 16 included studies with individual ratings and adapted scoring criteria. Appendix S2 details the standardized data extraction form used for systematic evidence synthesis. The complete study protocol is registered with INPLASY (International Platform of Registered Systematic Review and Meta-analysis Protocols) and is publicly available at: https://inplasy.com/inplasy-2025-8-0003/, accessed on 29 July 2025 (Registration No: INPLASY202580003, DOI: 10.37766/inplasy2025.8.0003).
