# Supplementary material for: Differential Impacts of Extreme Weather Events on Vector-Borne Disease Transmission Across Urban and Rural Settings: A Scoping Review
Source: Healthcare (Basel). 2025 Sep 25;13(19):2425. doi: 10.3390/healthcare13192425 (PMC12524810; doi:10.3390/healthcare13192425)
Supplement: Supplementary file 1 [file healthcare-13-02425-s001.zip › healthcare-3817675-supplementary.pdf]

Appendix S1. Complete Database Search Strategies

Search Parameters Applied Across All Databases

- **Date range:** 2000 to July 28, 2025
- **Final search completed:** July 28, 2025
- **Document types:** Article, Review
- **Language:** English
- **Deduplication method:** EndNote 21 (Clarivate Analytics, Philadelphia, PA, USA)
- **Duplicate removal process:** Automated duplicate detection followed by manual verification

PubMed Search Strategy (Final: 973 records)

Database: PubMed via NCBI
Initial results: 1,247 records
After filters applied: 973 records

Search Query:

```
("climate change"[MeSH Terms] OR "climate change"[Title/Abstract] OR "global
warming"[Title/Abstract] OR "extreme weather"[Title/Abstract] OR "heat
wave"[Title/Abstract] OR drought[Title/Abstract] OR flood[Title/Abstract] OR
heatwave[Title/Abstract] OR "climate variability"[Title/Abstract] OR
"temperature extreme"[Title/Abstract] OR "heat island"[Title/Abstract] OR
ENSO[Title/Abstract] OR "La Nina"[Title/Abstract] OR "El
Nino"[Title/Abstract])

AND

("vector-borne diseases"[MeSH Terms] OR "vector borne"[Title/Abstract] OR
"vector-borne"[Title/Abstract] OR malaria[Title/Abstract] OR
dengue[Title/Abstract] OR chikungunya[Title/Abstract] OR Zika[Title/Abstract]
OR mosquito[Title/Abstract] OR arbovirus[Title/Abstract] OR "yellow
fever"[Title/Abstract] OR leishmaniasis[Title/Abstract] OR "West
Nile"[Title/Abstract] OR "Rift Valley fever"[Title/Abstract] OR "Aedes
aegypti"[Title/Abstract] OR "Aedes albopictus"[Title/Abstract] OR
Anopheles[Title/Abstract])

AND

(urban[Title/Abstract] OR rural[Title/Abstract] OR city[Title/Abstract] OR
cities[Title/Abstract] OR village[Title/Abstract] OR slum[Title/Abstract] OR
periurban[Title/Abstract] OR community[Title/Abstract] OR
communities[Title/Abstract] OR settlement[Title/Abstract] OR
geographic[Title/Abstract] OR spatial[Title/Abstract] OR
metropolitan[Title/Abstract] OR agricultural[Title/Abstract])

AND
```

```
(transmission[Title/Abstract] OR outbreak[Title/Abstract] OR
epidemic[Title/Abstract] OR incidence[Title/Abstract] OR
prevalence[Title/Abstract] OR surveillance[Title/Abstract] OR
burden[Title/Abstract] OR risk[Title/Abstract] OR
distribution[Title/Abstract] OR pattern[Title/Abstract])
```

Filters Applied:

- Publication date: 2000/01/01 to 2025/07/28
- Article types: Journal Article, Review
- Languages: English
- Species: Humans

EMBASE Search Strategy (Final: 778 records)

Database: EMBASE via Ovid
Initial results: 3,012 records
After filters applied: 778 records

Search Query:

```
1. exp climate change/ OR exp global warming/ OR "climate change".ti,ab. OR
"global warming".ti,ab. OR "extreme weather".ti,ab. OR "heat wave".ti,ab. OR
drought.ti,ab. OR flood.ti,ab. OR heatwave.ti,ab. OR "climate
variability".ti,ab. OR "temperature extreme".ti,ab. OR "heat island".ti,ab.
OR ENSO.ti,ab. OR "La Nina".ti,ab. OR "El Nino".ti,ab.

2. exp vector borne disease/ OR exp malaria/ OR exp dengue/ OR exp
chikungunya/ OR exp Zika virus infection/ OR exp mosquito/ OR "vector
borne".ti,ab. OR "vector-borne".ti,ab. OR malaria.ti,ab. OR dengue.ti,ab. OR
chikungunya.ti,ab. OR Zika.ti,ab. OR mosquito.ti,ab. OR arbovirus.ti,ab. OR
"yellow fever".ti,ab. OR leishmaniasis.ti,ab. OR "West Nile".ti,ab. OR "Rift
Valley fever".ti,ab. OR "Aedes aegypti".ti,ab. OR "Aedes albopictus".ti,ab.
OR Anopheles.ti,ab.

3. exp urban health/ OR exp rural health/ OR urban.ti,ab. OR rural.ti,ab. OR
city.ti,ab. OR cities.ti,ab. OR village.ti,ab. OR slum.ti,ab. OR
periurban.ti,ab. OR community.ti,ab. OR communities.ti,ab. OR
settlement.ti,ab. OR geographic.ti,ab. OR spatial.ti,ab. OR
metropolitan.ti,ab. OR agricultural.ti,ab.

4. exp disease transmission/ OR exp disease outbreak/ OR exp epidemic/ OR
transmission.ti,ab. OR outbreak.ti,ab. OR epidemic.ti,ab. OR incidence.ti,ab.
OR prevalence.ti,ab. OR surveillance.ti,ab. OR burden.ti,ab. OR risk.ti,ab.
OR distribution.ti,ab. OR pattern.ti,ab.

5. 1 AND 2 AND 3 AND 4
```

Filters Applied:

- Publication year: 2000-2025
- Document type: Article, Review
- Language: English
- Human studies

Web of Science Search Strategy (Final: 2,351 records)

Database: Web of Science Core Collection
Initial results: 2,548 records
After filters applied: 2,351 records

Search Query:

```
TS=("climate change" OR "global warming" OR "extreme weather" OR "heat wave"
OR drought OR flood OR heatwave OR "climate variability" OR "temperature
extreme" OR "heat island" OR ENSO OR "La Nina" OR "El Nino")

AND

TS=("vector borne" OR "vector-borne" OR malaria OR dengue OR chikungunya OR
Zika OR mosquito OR arbovirus OR "yellow fever" OR leishmaniasis OR "West
Nile" OR "Rift Valley fever" OR "Aedes aegypti" OR "Aedes albopictus" OR
Anopheles)

AND

TS=(urban OR rural OR city OR cities OR village OR slum OR periurban OR
community OR communities OR settlement OR geographic OR spatial OR
metropolitan OR agricultural)

AND

TS=(transmission OR outbreak OR epidemic OR incidence OR prevalence OR
surveillance OR burden OR risk OR distribution OR pattern)
```

Filters Applied:

- Timespan: 2000-2025
- Document Types: Article, Review Article
- Language: English
- Research Areas: Public Environmental Occupational Health, Environmental Sciences, Infectious Diseases, Tropical Medicine, General Internal Medicine

---

Scopus Search Strategy (Final: 2,391 records)

Database: Scopus via Elsevier
Initial results: 2,944 records
After filters applied: 2,391 records

Search Query:

```
TITLE-ABS-KEY("climate change" OR "global warming" OR "extreme weather" OR
"heat wave" OR drought OR flood OR heatwave OR "climate variability" OR
"temperature extreme" OR "heat island" OR ENSO OR "La Nina" OR "El Nino")

AND

TITLE-ABS-KEY("vector borne" OR "vector-borne" OR malaria OR dengue OR
chikungunya OR Zika OR mosquito OR arbovirus OR "yellow fever" OR
leishmaniasis OR "West Nile" OR "Rift Valley fever" OR "Aedes aegypti" OR
"Aedes albopictus" OR Anopheles)

AND

TITLE-ABS-KEY(urban OR rural OR city OR cities OR village OR slum OR
periurban OR community OR communities OR settlement OR geographic OR spatial
OR metropolitan OR agricultural)

AND

TITLE-ABS-KEY(transmission OR outbreak OR epidemic OR incidence OR prevalence
OR surveillance OR burden OR risk OR distribution OR pattern)
```

Filters Applied:

- Publication year: 2000-2025
- Document type: Article, Review
- Language: English
- Subject areas: Medicine, Environmental Science, Agricultural and Biological Sciences, Social Sciences

Table S1. Database Search Results and Strategies

Database	Initial Results	After Filters Applied	Final Included
**PubMed**	**1,247**	**973**	**Search strategy in Appendix S1**
**EMBASE**	**3,012**	**778**	**Search strategy in Appendix S1**
**Web of Science**	**2,548**	**2,351**	**Search strategy in Appendix S1**
**Scopus**	**2,944**	**2,391**	**Search strategy in Appendix S1**
**Total**	**9,751**	**6,493**	**After deduplication: 4,875**
**Final Screening**			**16 studies included**

Filters applied: Publication years 2000-2025, Document types (Article, Review), Language (English)

Table S2. Quality Assessment of All 16 Included Studies Using Adapted Quality Criteria

Study	Study Type	Assessment Method	Selection/Design (0-4)	Comparability/Validity (0-2)	Outcome/Results (0-3)	Total (0-9)	Quality Rating	Notes
**Temperature and Vector Dynamics**								
**Brady et al.**	Global modeling/laboratory	Adapted criteria for modeling studies	4★★★★	2★★	3★★★	9/9	High	Comprehensive global analysis, multiple validation approaches
**Agyekum et al.**	Laboratory experiment	Adapted criteria for experimental studies	4★★★★	2★★	3★★★	9/9	High	Well-controlled design, multiple life stage parameters
**Ciota et al.**	Laboratory experiment	Adapted criteria for experimental studies	4★★★★	2★★	3★★★	9/9	High	Multiple species comparison, temperature gradient design
**Tesla et al.**	Laboratory/modeling	Adapted criteria for experimental studies	4★★★★	2★★	3★★★	9/9	High	Rigorous temperature-controlled virus replication study
**Extreme Weather Events and Disease Outbreaks**								
**Caillouët et al.**	Surveillance study	Newcastle-Ottawa Scale (cohort)	3★★★	1★	2★★	6/9	Moderate	Post-hurricane surveillance, limited temporal scope
**Shaman et al.**	Surveillance study	Newcastle-Ottawa Scale (cohort)	3★★★	2★★	3★★★	8/9	High	Long-term drought analysis, well-documented methodology
**Danforth et al.**	Laboratory experiment	Adapted criteria for experimental studies	4★★★★	2★★	3★★★	9/9	High	Controlled temperature cycling experiment
**Mourelatos et al.**	Surveillance study	Newcastle-Ottawa	3★★★	1★	2★★	6/9	Moderate	Flood-associated

		Scale (cohort)						transmission study, limited follow-up
**Geographic Range Expansion and Altitude Shifts**								
**Siraj et al.**	Surveillance study	Newcastle-Ottawa Scale (cohort)	4★★★★	2★★	3★★★	9/9	High	Multi-country altitude analysis, long-term data
**Colón-González et al.**	Climate modeling	Adapted criteria for modeling studies	4★★★★	2★★	3★★★	9/9	High	Multi-GCM approach, uncertainty quantification
**González et al.**	Ecological modeling	Adapted criteria for modeling studies	3★★★	2★★	2★★	7/9	Moderate-High	Species distribution modeling, climate scenarios
**Disease Burden and Geographic Distribution**								
**Alvar et al.**	Systematic review	Adapted criteria for reviews	4★★★★	1★	2★★	7/9	Moderate-High	Comprehensive global data compilation
**Li et al.**	Surveillance study	Newcastle-Ottawa Scale (cohort)	3★★★	2★★	3★★★	8/9	High	Spatiotemporal analysis, mosquito-climate relationships
**Vector Ecology and Environmental Factors**								
**Gachoki et al.**	Satellite modeling	Adapted criteria for modeling studies	4★★★★	2★★	3★★★	9/9	High	Satellite-based habitat identification, field validation
**Konan et al.**	Field study	Newcastle-Ottawa Scale (cross-sectional)	3★★★	1★	2★★	6/9	Moderate	Urban forest ecology study, limited temporal data
**Lindström et al.**	Laboratory experiment	Adapted criteria for experimental studies	3★★★	2★★	2★★	7/9	Moderate-High	Species-specific hatching responses to flooding

Adapted Quality Assessment Criteria:

For Laboratory Experiments (Selection/Design 0-4):

- Experimental design rigor (randomization, controls)
- Sample size adequacy
- Temperature/climate exposure measurement
- Replication and statistical power

For Modeling Studies (Selection/Design 0-4):

- Data quality and sources
- Model validation approaches
- Spatial/temporal resolution
- Uncertainty assessment

For Observational Studies (Newcastle-Ottawa Scale):

- Standard NOS criteria for cohort/cross-sectional studies
- Exposure ascertainment
- Outcome assessment
- Follow-up adequacy

Quality Distribution Summary (n=16):

- High Quality (7-9 stars): 11 studies (69%)
- Moderate-High Quality (6-7 stars): 3 studies (19%)
- Moderate Quality (4-5 stars): 2 studies (12%)
- Low Quality (0-3 stars): 0 studies (0%)

Mean Quality Score: 7.9/9

Study Type Distribution:

- Laboratory experiments: 6 studies
- Surveillance/observational studies: 5 studies
- Modeling studies: 4 studies
- Systematic review: 1 study

Note: Quality assessment employed study-type-specific criteria. Observational studies were evaluated using the Newcastle-Ottawa Scale, while laboratory experiments and modeling studies were assessed using adapted criteria focusing on experimental design rigor, data quality, and validation approaches appropriate to each study type.

Appendix S2. Data Extraction Form

Variable Category	Specific Data Fields	Data Type/Format
**Study Identification**	Author(s)	Text
	Publication year	Numeric (YYYY)
	Title	Text
	Journal	Text
	DOI/PMID	Text
**Study Characteristics**	Study design	Categorical (Cross-sectional, Longitudinal, Case-control, etc.)
	Study duration	Numeric (months/years)
	Sample size	Numeric
	Study setting	Categorical (Urban, Rural, Mixed, Peri-urban)
	Data collection period	Date range (YYYY-YYYY)
**Geographic Information**	Country	Text
	Region/Province	Text
	Urban/rural classification	Categorical (Urban, Rural, Peri-urban, Mixed)
	Climate zone	Categorical (Tropical, Temperate, Arid, etc.)
	Geographic coordinates	Numeric (if available)
**Population Characteristics**	Age range	Numeric/Text
	Gender distribution	Percentage
	Socioeconomic indicators	Text/Categorical
	Population density	Numeric (per km^2)
	Vulnerability factors	Text
**Climate Exposures**	Temperature variables	Numeric (°C, temperature ranges)
	Precipitation patterns	Numeric (mm, seasonal patterns)
	Extreme weather events	Categorical (Drought, Flood, Heatwave, Storm)
	Climate data source	Text
	Temporal resolution	Categorical (Daily, Monthly, Seasonal, Annual)
**Vector-Borne Disease Outcomes**	Disease type	Categorical (Malaria, Dengue, Chikungunya, Zika, etc.)
	Incidence rates	Numeric (cases per population)
	Transmission metrics	Numeric (R0, transmission rates)
	Vector species	Text
	Disease surveillance data	Text/Numeric

**Urban-Rural Comparisons**	Differential impacts	Text (Qualitative description)
	Transmission pathways	Text (Infrastructure vs. ecosystem-mediated)
	Risk factors by setting	Text
	Comparative outcomes	Numeric/Text
	Settlement-specific findings	Text
**Mechanistic Pathways**	Vector ecology factors	Text
	Infrastructure influences	Text
	Environmental mediators	Text
	Socioeconomic mediators	Text
	Pathway mechanisms	Text
**Adaptation Strategies**	Intervention types	Categorical/Text
	Prevention measures	Text
	Policy recommendations	Text
	Implementation setting	Categorical (Urban, Rural, Both)
	Effectiveness indicators	Numeric/Text
**Study Quality Indicators**	Methodology rating	Categorical (High, Moderate, Low quality)
	Data sources quality	Text
	Study limitations	Text
	Risk of bias assessment	Categorical/Text
	Generalizability	Text
